# A “thermodynamic” model of synergistic interaction of human elbow flexor muscles during simultaneous change in their length and force

**DOI:** 10.3389/fphys.2026.1904516

**Published:** 2026-09-22

**Authors:** Alexander Kostyukov, Andriy Gorkovenko, Elena Kolosova

**Affiliations:** Department of Movement Physiology, Bogomoletz Institute of Physiology, National Academy of Sciences of Ukraine, Kyiv, Ukraine

**Keywords:** central motor commands, electromyogram, muscle hysteresis, muscle synergy, single-joint movements

## Abstract

**Introduction:**

Muscle hysteresis reflects nonlinear changes in electromyographic (EMG) activity during isolated changes in the length and force of individual muscles. However, previous studies have primarily considered changes in individual muscle state variables, whereas the hysteresis behavior associated with *simultaneous* changes in muscle length and force, remains insufficiently understood. Therefore, this study aimed to develop a mathematical model of EMG responses to simultaneous changes in muscle force and length using population-averaged experimental data from the authors’ previously published study. Characterizing the history-dependent relationship among muscle activation, force, and length may contribute to adaptive control strategies for robot-aided rehabilitation, with potential applications in the assessment of motor dysfunction and rehabilitation personalization.

**Methods:**

A mathematical model was developed to describe the intensity of EMG signals from human elbow flexor muscles during *simultaneous* changes in muscle force and length. EMG signals and muscle state parameters were obtained from ten participants during a combination of externally controlled joint movements and active force generation under visual biofeedback. The framework of time-averaged derivatives of muscle state parameters was used to derive algorithms for estimating the activation intensity of synergistic muscles during motor tasks.

**Results:**

The T-model (“thermodynamic” model) was developed based on the functional interdependence of three key parameters: muscle length, force, and the intensity of incoming activation. Algorithms were proposed to predict the activation intensity of synergistic muscles performing motor tasks involving simultaneous changes in muscle force and length. The results show that the use of time-averaged derivatives of muscle state parameters provides a close approximation of central motor commands, with COD values ≥0.9389 across the tested conditions. The analysis demonstrates that the amplitude of hysteresis effects increases with *coinciding* changes in muscle force and length and decreases when these changes are *opposing*.

**Conclusion:**

The modeling results demonstrate that hysteresis in agonist muscle groups is strongly dependent on the interaction between *simultaneous* changes in muscle force and length. These findings highlight the importance of considering coupled force–length dynamics in the analysis of neuromuscular control and provide a basis for further applications of muscle hysteresis analysis in motor control and rehabilitation.

## 1 Introduction

Within the framework of dynamic systems theory, skeletal muscle can be considered as a nonlinear dynamic system defined by three state variables: length (L), force (F), and the intensity of the incoming activation (E). Based on this simplification, it is possible to formulate the existence of three separate functional dependencies defining each of the above-mentioned variables as a function of the other two: L(E, F), F(E, L), and E(L, F). The first two functions have clear priority in the analysis and modeling of muscle dynamics (Hill, 1953; Huxley, 1974; Herzog, 2014; Proske et al., 2014). These relationships describe the nonlinear interaction between muscle length, force, and activation and therefore provide a basis for characterizing the inherently nonlinear behavior of skeletal muscle. It could be assumed that the steady state nonlinear effects followed by changes of activation intensity may also differ from the after-effects appeared due to isolated changes of the mechanical parameters.

The L(E, F), F(E, L) dependencies were predominantly investigated in experiments on isolated muscle fibers and different modifications of the muscle-nerve preparations. One can point out here classical studies of Edman, 1975; Edman et al., 1978; Rack, 1966; Rack and Westbury, 1969, 1974. Some of nonlinearities in functional dependencies L(E, F), F(E, L) were identified in cat experiments with method of distributed frequency-modulated stimulation of efferents, which allowed imitate a natural income of the activation neural signals in the supplying efferent fibers. These experiments demonstrated pronounced nonlinearities, including the well-known movement history-dependent hysteresis, where muscles generate higher forces during lengthening than during shortening, and these differences persist after movement termination (Kostyukov and Korchak, 1998; Proske et al., 2014; Fukutani et al., 2019). Similar nonlinearities emerge when assessing L(E, F) relationships under isotonic conditions, what has been shown in nerve-muscle experiments with studying transients under abrupt transitions from isometry to isotony conditions of contraction (Rack and Westbury, 1974; Kostyukov, 1987). Powerful hysteresis effects reflecting intrinsic contractile properties were identified for F(E, L) dependency during controlled stretching-shortening cycles of muscle contraction (Kostyukov, 1987, 1998; Proske et al., 1992; Fukutani et al., 2019; Pinnell et al., 2019). Most prior investigations analyzed these dependencies by varying only one variable whereas holding the others constant (Minetti, 1994; Herzog, 2014; Proske et al., 2014; Herzog, 2018). Regarding the E(L, F) relationship, it should be noted that there are numerous experimental and theoretical studies examining EMG patterns in real movement programs. Additionally, it is useful to note works of Tal'nov et al. (1997, 1999), who studied averaged EMGs in series of standard slow flexion movements using joint angle as a controlled parameter. The exponential EMG shape described in the study directly points to nonlinear effects in central commands, revealing areas of different static sensitivity of the elbow flexor muscles depending on their length.

In spite of absence of direct relationships between the muscle state parameters, which might be present in the assumed E(L, F) dependencies, such studies allow obtaining a real evaluation of the activation intensities signals coming to synergic muscle groups in really controlled test movements. In recent years, traditional experiments used to assess the nature of EMG intensity changes during various test movements have been improved by applying a new methodological approach that allows more precise control over the length of the synergistic muscles under study and the forces they generate. This method consists in independent control of changes in muscle lengths and their forces during human arm movements (Gorkovenko et al., 2020b). A robotic mechatronic device (RMD) creates a programmed platform movement by positioning the participant’s hand along a predetermined trajectory. Using visual biofeedback, the participant performs another predetermined action, creating a specific force vector by pressing a vertical rod located on the platform and containing a force vector sensor. Muscle lengths and forces trajectories are programmed using methods based on preliminary biomechanical modeling of bones and muscles. This approach has already been applied to circular two-joint movements and to linear cyclic changes in elbow flexor length combined with controlled force generation (Kostyukov et al., 2022; Gorkovenko et al., 2025, 2026).

The non-linear properties of muscle contraction have been studied for over a century (Tomalka, 2023). The term “hysteresis” encompasses a wide range of nonlinear effects in motor control systems. In the literature, elements of muscle hysteresis are often referred to as “residual force/torque enhancement/depression” (RFE, RFD). Physiologically, RFE manifests as the muscle developing a noticeably greater final force when subjected to eccentric active stretching (Herzog et al., 2006). At the same time, it is worth noting that research into the RFE and RFD phenomena has mainly focused on the study of eccentric muscle movement, overlooking the fact that in everyday life, muscles typically operate in a cyclic mode, where elongation and contraction are repeated in large numbers. This fact explains why the word hysteresis is virtually never mentioned in the literature dedicated to RFE and RFD. It should be noted that in the cyclic mode of muscle work, the classical manifestation of muscle hysteresis is recorded in the form of corresponding hysteresis loops E(L) or E(F). One of the objectives of this article is an attempt to build a bridge between the study of the RFE and RFD phenomena and the muscle hysteresis loops.

The present study provides a further theoretical analysis of the recently published experiments describing central motor commands to the elbow flexor muscles with simultaneous changes in force and length (Gorkovenko et al., 2026), using as a basis the so-called T-model (“thermodynamic” model) developed previously (Kostyukov, 2019; Kostyukov et al., 2022). The study by Gorkovenko et al. (2026) used a methodological approach first proposed by Zasada et al. (2020), which consisted of a complete separation of two independent motor programs: 1) the movement itself, which is specified externally by RMD; 2) the generation of force, which the subject need to perform independently in the biofeedback mode. The external motion control program eliminates the commonly used joint angle input; instead, due to realistic modeling of muscle-bone connections, muscle lengths can be directly selected as input signals; similarly, precisely calculated muscle forces are used instead of respective torque values (Gorkovenko et al., 2020a, 2020b). It is precisely thanks to these methodological prerequisites that a real opportunity arose to compare the actual values ​​of activation intensity and mechanical parameters of movements in different subjects. The next step was to apply the normalization procedure to all experimental recordings obtained from different subjects, which allowed finally to use an effective averaging method over the entire set of compared signals in a sufficiently large group of subjects (method of population averaging) (Gorkovenko et al., 2026).

The use of more engaging experimental paradigms, such as virtual reality (VR), may further enhance participant motivation and involvement during repetitive motor tasks. VR-based and task-oriented approaches have also shown potential for integrating EMG and muscle-synergy information into myocontrol and robotic rehabilitation strategies, suggesting a promising direction for future studies of muscle hysteresis and motor control (Buongiorno et al., 2020).

Beyond its fundamental relevance to the understanding of muscle mechanics and motor control, muscle hysteresis analysis may have potential clinical applications in the assessment of neuromuscular function and motor impairment. In particular, a more accurate description of the history-dependent relationship among muscle activation, force, and length may provide additional information about alterations in motor control that are not captured by conventional analyses of EMG amplitude alone. Such information could potentially contribute to the development of more physiologically informed models of human movement and support the design of adaptive control strategies in robot-aided rehabilitation, including assist-as-needed approaches in which robotic assistance is adjusted according to the patient’s voluntary motor output. Previous studies have demonstrated the potential of EMG-driven and adaptive control strategies for regulating robotic assistance during rehabilitation (Buongiorno et al., 2015, 2018). From this perspective, incorporating hysteresis-related characteristics into models of synergistic muscle activation may provide an additional dimension for characterizing the temporal and mechanical context of neuromuscular activation, opening up new possibilities for dysfunction assessment and personalization of robotic rehabilitation.

Hypothesis. The authors propose representing the central commands sent to human muscles during various movement patterns as nonlinear surfaces E(L, F), defining averaged EMG intensities (E) with respect to orthogonally located parameters of muscle length (L) and force (F). This creates an analogy with classical thermodynamics, which uses averaged first-order derivatives of system state parameters to simplify the analysis of nonlinear effects in complex molecular systems. This study presents a similar attempt to analyze EMG intensity during simultaneous changes in muscle length and force using averaged first-order derivatives over chains of limited movement areas.

Study Structure. The paper is divided into Sections 1–4 as standard. Since it contains a theoretical discussion and mathematical modeling of the authors previously published experimental results, Section 2 (Methods) provides only a brief description of the methods used to obtain the simulated experimental recordings. Sections 3.1 and 3.2 contain the general mathematical formulation of the model and a graphical description of the original experimental recordings and the simulation results. Sections 3.3 and 3.4 provide a visual display for comparing the main elements of the model, including the first derivatives of force, length, and EMG, which were calculated using the corresponding normalized population-averaged curves. Section 3.5 describes the inverse problem for the T-model, which allows to estimate its important components: activation compliance and muscle stiffness. Section 3.6 provides a general formulation of the T-model and describes statistical methods for quantifying the piecewise linear approximation of averaged EMG recordings using the T-model.

## 2 Materials and methods

The experimental methodology is described in detail in the previous study (Gorkovenko et al., 2026). Only the main points will be presented here. Ten healthy, right-handed participants (6 males and 4 females, aged 26.6 ± 5.4 (mean ± SD) years) took part in the study. Each participant was required to perform 10 tests, each consisting of 10 repetitions of the corresponding motor tasks. Each trial lasted approximately 1 minute, with 10–15 second rest intervals between successive trials. The task required the participant to generate a specific force with the hand in the horizontal plane during passive arm movement, with the force parameters monitored via visual biofeedback. The passive arm movement was driven by a specialized RMD (Zasada et al., 2020). The separate tests included two independent parts: the externally imposed joint movements provided by RMD, in parallel, the participants produced voluntary forces controlled through visual biofeedback. No signs of fatigue were observed during the experiment or immediately after its completion.

The surface EMGs were recorded from three elbow flexor agonist muscles of each subject (*m. brachioradialis, m. biceps brachii, caput breve et caput longum*); as well as the position coordinates of the hand in the horizontal plane of the operational space and the force generated by the participant’s hand. EMG records in each subject were amplified with a band-pass filtering of 10–5,000 Hz for each channel, subsequently full-wave rectified, low-pass Butterworth filtered at 10 Hz, and averaged over ten repetitions of each movement test (Suglia et al., 2026). The parameters of joint angle and hand force were set in such a way that the length and force of the muscles varied in a specific manner. Crucially, the length and force of the aforementioned muscle could change either in the same or in opposite direction. The maximum muscle force amplitude had two levels: full and half. The isotonic mode of muscle operation was investigated in two additional tests.

For comparing the movement patterns and EMGs in different participants, normalized recordings of L(t), F(t), and E(t) were used. The scale of each of the normalized signals was set at a level at which their integrals over the testing period were taken to be equal to one. The normalized averaged EMG signals were further averaged within a group of three elbow flexor muscles for each subject, and the resulting signals were subjected to an inter-group averaging procedure for ten subjects (Gorkovenko et al., 2026).

## 3 Results

### 3.1 Basic formulation of T-model

The T-model is based on the use of functional interdependence of three key parameters that determine the sustained force generated by a muscle, depending on its length and the intensity of the incoming activation. The activation intensity should be considered as an integrated estimation of the flow of impulse activity arriving along all efferent fibers belonging to the muscle motor units. In a real experiment, such an estimate can be obtained using averaged recordings of EMG activity from the skin surface over the muscle under study. Such a functional dependence of three values, E (activation intensity), L (muscle length) and F (muscle force), can be considered, in particular, as a certain nonlinear surface F(E, L), determined by a set of two regular sequences of perpendicular sections with constant values ​​of muscle length and activation intensity:

(1) $$dF(E,L)={\frac{\partial F}{\partial E}|}_{L=const}dE+{\frac{\partial F}{\partial L}|}_{E=const}dL$$

The domain of definition of the two-variable function F(E, L) is mainly limited by purely physiological and biomechanical factors. The simplified form of Equation 1 can be rewritten as follows:

(2) $$dF(E,L)=\frac{\partial F}{\partial E_{L}}dE+\frac{\partial F}{\partial L_{E}}dL$$

By analogy with the formalism used in classical thermodynamics, the differentials dF, dE, and dL in Equation 2 will be further considered as exact differentials (Thomas and Finney, 1996). Thus, this equation can also be considered as a basis for describing active muscle contractions within the framework of the previously presented T-model (Kostyukov, 2019).

(3) $$dE=\frac{\partial E}{\partial F_{L}}(dF-\frac{\partial F}{\partial L_{E}}dL)=C_{L}^{a}\left(dF-S_{E}^{m}dL\right)$$

In addition, one obvious notation will be introduced for the corresponding derivative in Equation 3.: $\frac{\partial F}{\partial L_{E}}=S_{E}^{m}$, muscle stiffness, and it is obvious that the reciprocal of the stiffness will indicate the muscle compliance: $\frac{\partial L}{\partial F_{E}}=C_{E}^{m}$. These designations coincide with standard terms of muscle physiology, therefore, by analogy with the designations given above, additionally two terms: $\frac{\partial E}{\partial L_{F}}=S_{F}^{a}$ “activation stiffness”, and $\frac{\partial E}{\partial F_{L}}=C_{L}^{a}$ “activation compliance”, will be introduced. The meaning of these terms also seems obvious.

Using the above introduced terms, the following expression can be derived, it defines the relationship between the time derivatives of the signals under study:

(4) $$\frac{\partial E}{\partial t}=C_{L}^{a}\frac{\partial F}{\partial t}-C_{L}^{a}S_{E}^{m}\frac{\partial L}{\partial t}$$

A special interest may present a case of isotonic regime related to a constancy of the force:

(5) $$\frac{\partial E}{\partial t}=-C_{L}^{a}S_{E}^{m}\frac{\partial L}{\partial t}$$

Thus, Equation 5 represents the opposite directions of change in activation intensity and muscle length for the isotonic regime.

For relatively short interval of force and length change, let it will be further signified as ΔT, it is possible to integrate both sides of Equation 4:

(6) $$\int_{\Delta T}\frac{\partial E}{\partial t}dt=\int_{\Delta T}C_{L}^{a}\frac{\partial F}{\partial t}dt-\int_{\Delta T}C_{L}^{a}S_{E}^{m}\frac{\partial L}{\partial t}dt$$

Assuming a relative constancy of $S_{F}^{a}$ and $C_{L}^{a}$ over the interval ΔT and using the corresponding average values ​​of the force and length derivatives over this interval, a linear approximation of the integral form given by Equation 6 can be obtained as follows:

(7) $$\bar{\frac{\partial E}{\partial t}}=C_{L}^{a}\bar{\frac{\partial F}{\partial t}}-C_{L}^{a}S_{E}^{m}\bar{\frac{\partial L}{\partial t}}=C_{L}^{a}(\bar{\frac{\partial F}{\partial t}}-S_{E}^{m}\bar{\frac{\partial L}{\partial t}})$$

where the lines above the derivatives indicate the averaging procedure in the corresponding interval ΔT.

The averaging procedure occurred to be especially effective when the average force and length derivatives $\bar{\frac{\partial F(\tau )}{\partial \tau }}$and $\bar{\frac{\partial L(\tau )}{\partial \tau }}$ do not change over these time intervals. Then, according to Equation 7, it follows a constancy for the average value for the activation intensity derivative $\bar{\frac{\partial E(\tau )}{\partial \tau }}$. Provided that the initial conditions for the activation intensity and its average derivative $\bar{\frac{\partial E(\tau )}{\partial \tau }}$ over the time interval ΔT are known, the following expression will give a linear approximation of *E*(t) over this interval:

(8) $$\tilde{E(t)}=E(t_{0})+\int_{t_{0}}^{t_{0}+\Delta T}\bar{\frac{\partial E(\tau )}{\partial \tau }}d\tau$$

Equation 8 represents a procedure for piecewise linear approximation of the activation intensity *E(t)* during the minimum time interval ΔT in which its average derivative $\bar{\frac{\partial E(\tau )}{\partial \tau }}$ can be relatively stable. Clearly, three directions of change in *E(t)* are possible within a given time interval: growth, stabilization, and decline. These possibilities can be assessed by determining the average value of the activation intensity derivative. On the other hand, choosing the correct time interval for the approximation procedure is of great importance. If the derivative changes repeatedly within the interval, this may indicate that the interval was chosen too large, and the quality of the approximation will be poor.

Despite somewhat trivial appearance of Equation 8, it can be quite useful if it is possible to divide a sufficiently long period of analysis into a chain of time intervals during which the corresponding average derivative of the activation intensity can be separately estimated. Similarly, based on Equation 8, a more detailed expression can be obtained for the linear approximation of the activation intensity in the time interval ΔT, where there are correct estimates of the average derivatives of the force and length:

(9) $$\tilde{E(t)}=E(t_{0})+\int_{t_{0}}^{t_{0}+\Delta T}C_{L}^{a}(\bar{\frac{\partial F}{\partial t}}-S_{E}^{m}\bar{\frac{\partial L}{\partial t}})d\tau$$

It is important to correctly determine the initial conditions $E(t_{0})$ for Equations 8, 9.

### 3.2 Piecewise linear approximation of the averaged EMG records

Below, it will be considered the application of the above approach to modeling and additional analysis of some experimental results. This model will be applied to the records presenting a purely theoretical averaging of similar experimental results obtained in this study within a group of ten subjects. **Figure 1** shows the results of such averaging of the EMG data of the elbow flexors, combined with theoretically reconstructed mechanical characteristics of the corresponding muscles, participating in identical movement tests of a group of ten subjects. The tests included movements with respective elbow joint angle changes in the range approximately between 45 and 100 degrees.

**Figure 1:**
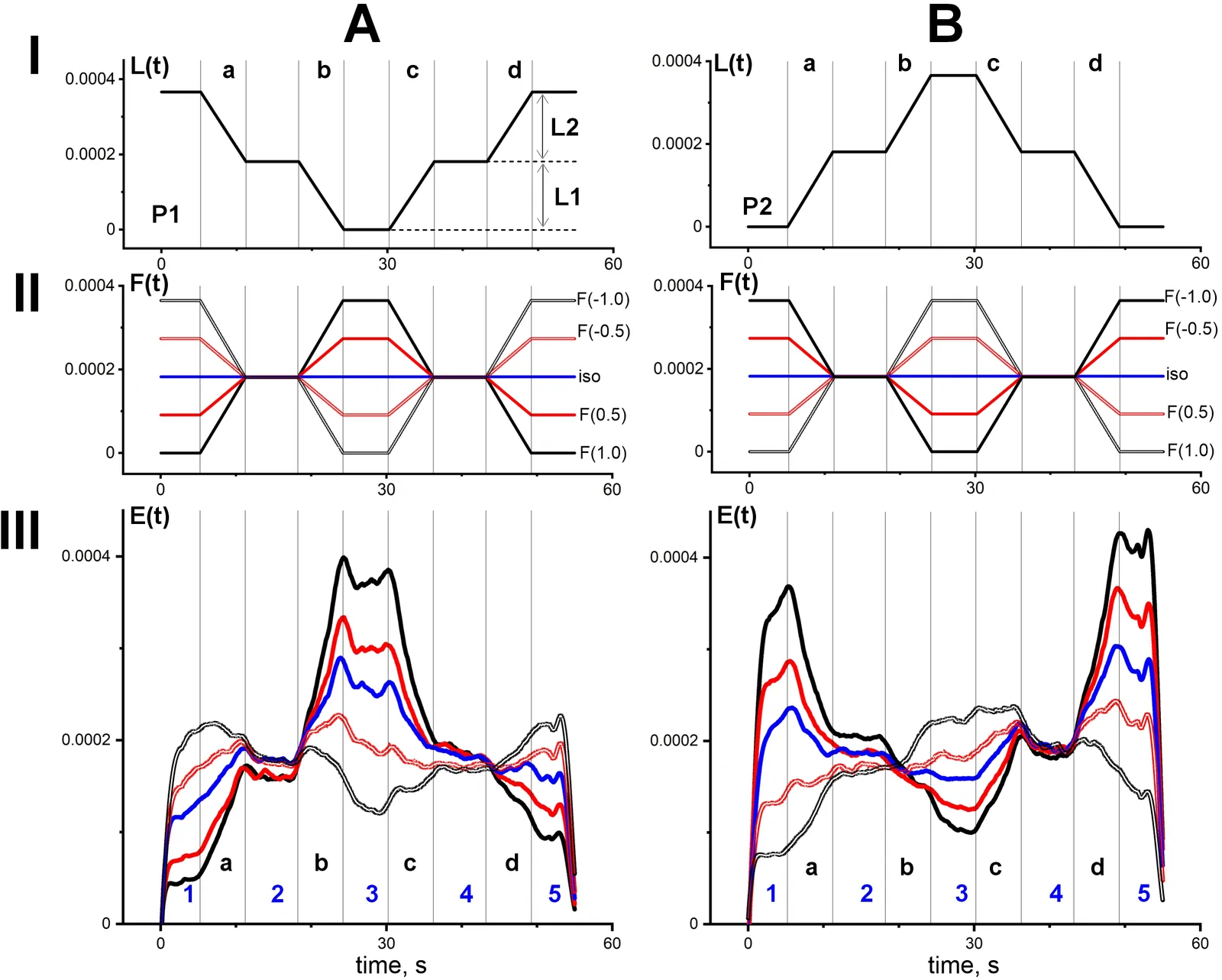
The proposed model is tested on that part of the experimental data from the study by Gorkovenko et al., 2026, which presents the results of the population averaging over a set of EMGs registered in group of ten subjects during identical hand movements. **(A, B)** Normalized recordings of muscle lengths L(t), acting forces F(t) and averaged EMG E(t), recorded respectively in combinations of two movement patterns P1 and P2 and five force patterns F(1.0), F(0.5), iso, F(-1.0), F(-0.5); additional normalization of the scales relative to those previously used (Gorkovenko et al., 2026) includes equating the area of ​​the studied curves to unity. Note the correspondence of colors and line structures between the sets of F(t) and E(t) curves. The time ranges with simultaneous linear changes in L(t) and F(t) are denoted by lowercase black letters a-d; in fact, the main analysis concerns precisely these sections of the corresponding trajectories. Blue numbers from 1 to 5 indicate time intervals corresponding to the constancy of mechanical parameters L(t) and F(t) in the tests.

The experiments shown in **Figure 1** used identical symmetrical amplitude-time profiles of muscle length and force, shaped as double trapezoids with the vertices pointing upward or downward; movement tests included two combinations of length changes P1, P2, each of which corresponded to five variations of force change, shown by corresponding lines of different colors and structures; between the five sections (1-5) with constant force and length values, four more sections (a-d) were placed where these parameters were varied.

When comparing EMG responses across five test types reflecting different changes in force within the two motor patterns P1 and P2, it is probably best to start by considering the isotonic movements, which are shown in **Figure 1** as blue lines. All changes in the activation intensity E(t), affecting the muscles under study through biofeedback mechanisms, in this case are mainly determined by changes in their length L(t), which are set externally by RMD. Despite the difference between tests P1 and P2, it is necessary to emphasize the actual coincidence of their halves: 1) a-b in P1 and c-d in P2; 2) c-d in P1 and a-b in P2. This coincidence is due to the use of symmetrical combinations of force changes in these experiments.

**Figure 2** shows the process of piecewise linear approximation of experimental records E(t) using Equation 8. As an example, process for the combination of test movements P1 with the implementation of the force pattern F(1.0) will be firstly considered; this combination demonstrates the interaction of oppositely directed changes in muscle force and length (**Figure 2A**). The E(t) record was then differentiated and the average values ​​of the derivatives were determined in the successive chain of time intervals CH: 1-a-2-b-3-c-4-d-5 (bars $\bar{\frac{\partial E}{\partial t}}$ are shown in blue and black for intervals 1–5 and a-d, respectively). Integration of the bar sequence with using Equation 8 can define the entire process of the piecewise linear approximation $\tilde{E(t)}$:

**Figure 2:**
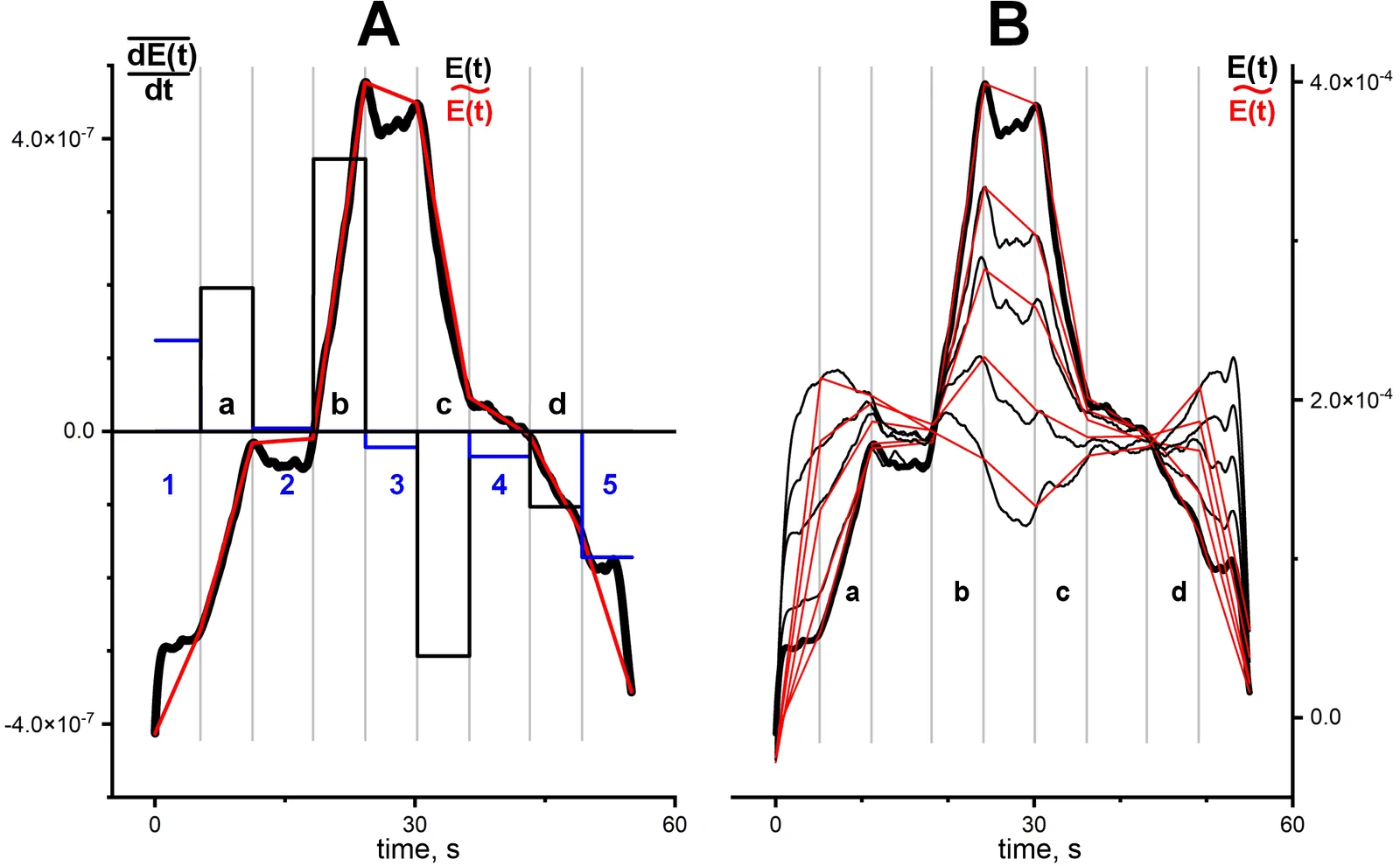
Procedure of piecewise linear approximation of the EMGs registered in tests with similar patterns of length and force changes. **(A)** Superposition of the following signals: 1) thick black line E(t), EMG signal shown by thick line in **Figure 1AIII**; 2) average derivatives $\bar{E(t)}$, sequence of blue and black vertical bars; 3) piecewise linear approximation $\tilde{E(t)}$, red line. **(B)** Overlay of all E(t) records in P1 tests (black) and the corresponding piecewise linear fit curves (red).

(10) $$\tilde{E(t)}=\int_{CH}\bar{\frac{\partial E}{\partial t}}dt$$

**Figure 2B** shows an overlay of all E(t) records in P1 tests for the five possible force changes, along with their approximations. The quality of the approximations in the time ranges a-d, where length and force changed simultaneously, appears to be quite high, but appears to depend on the amplitude and direction of the force and length changes. This is clearly visible, at least in the time range b, when comparing the upper and lower records of E(t), corresponding to the responses to forces of maximum amplitude F(1.0) and F(-1.0), guided in opposing and coinciding directions, respectively, relative to L(t). Moreover, the discrepancies between the E(t) signals and their approximations visually increase in intervals with constant values ​​of L(t) and F(t). At least in part, these discrepancies may be due primarily to residual effects accompanying the dynamic components of muscle contractions during the movement phases (intervals 2, 3, 4), or directly related to the strong influence of the onset and offset of activation (intervals 1, 5).

### 3.3 Comparison of time derivatives of signals E(t), F(t) and L(t)

The piecewise linear approximation of the E(t) records described above is based on the use of their time derivatives, so the detailed structure of the derivative curves themselves may be of interest. **Figure 3** shows the superpositions of the time derivatives defined for the signals E(t), F(t), and L(t), including all their combinations. It should be noted that the observed reactions of increase and decrease in derivatives $\frac{\partial E(t)}{\partial t}$are most pronounced in the phases of movement a-d, when the step changes in the derivatives of force and length are directed opposite to each other (**Figure 3**, rows I, II). At the same time, they remain noticeable during isotonic contractions (row III) and decrease, but do not disappear completely, in tests where changes in force and length coincide in direction (**Figure 3**, rows IV, V). Returning to the formal consideration, it is possible to conclude that derivatives $\frac{\partial E(t)}{\partial t}$ predominantly counteract the movement velocity signal $\frac{\partial L(t)}{\partial t}$, whereas adding a force derivative $\frac{\partial F(t)}{\partial t}$ reduces this counteraction, in some cases even causing a reverse change in the resulting response. Alternatively, one could speculate that the “predominance” of length changes over force changes observed in this experiment is simply determined by their actual ratio, so that increasing force changes would have the opposite effect. Given the general characteristics of the derivatives $\frac{\partial E(t)}{\partial t}$ located outside the regions of length and/or force changes, and excluding the initial and final phases of the motor trials (time intervals 1 and 5), one could conclude that they fluctuated primarily randomly around zero, naturally exhibiting some initial deviations after the preceding regions of length and/or force changes.

**Figure 3:**
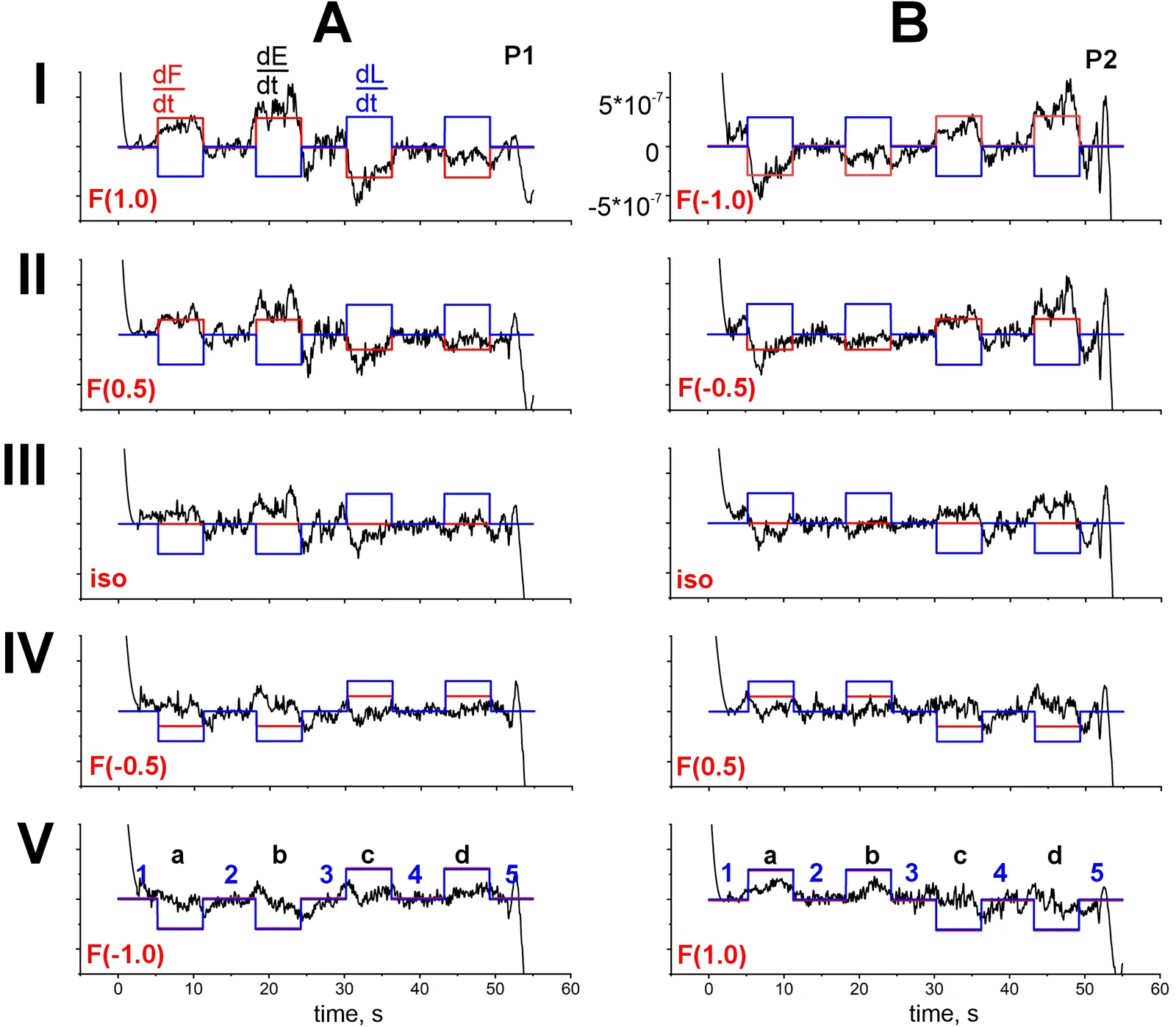
Comparison of actual values ​​of time derivatives determined for average values ​​of activation intensity E(t); muscle force F(t); muscle length L(t). **(A, B)** The derivatives were determined based on the corresponding records presented for the P1 and P2 movement tests in **Figure 1**. Due to the preliminary normalization procedure applied to the signals, their time derivatives allow them to be presented on the same scale. The corresponding isotonic contractions are shown in row III, reactions with opposite and coinciding ratios of changes in force and length are depicted in rows (I, II) and (IV, V), respectively. The corresponding derivatives are distinguished by color, as shown in panel **AI**, note a complete coinciding of the force and length derivatives in line V.

### 3.4 Statistical analysis of the time derivatives of the EMG signals

It is likely that the nature of derivative changes $\frac{\partial E(t)}{\partial t}$ within the time zones where muscle length and force vary may be important for analyzing the entire *E(t)* record. **Figure 4** shows these changes for forces with maximum amplitude (row III). Firstly, one can notice a clear difference between the amplitudes and signs of these derivative records in different parts of the tests. When comparing the derivatives in different parts of the P1 and P2 movement tests, a clear dependence of their signs on the direction of length change is evident. The derivatives are positive (negative) in the first (second) halves of the P1 tests, whilst in the P2 tests these signs are reversed. Thus, the signs of the derivatives are positive during muscle shortening and negative during muscle lengthening, what corresponds to Equations 7–9 for cases of opposite directions of force and length change. On the other hand, the clear difference between the amplitudes of the derivatives in adjacent phases of the movements may be somewhat striking, despite the equality of movement velocities. However, this can be qualitatively explained by linking this observation with previously obtained data on the dynamics of EMG intensity in the elbow flexor muscles during slow isotonic movements of the elbow joint in the direction of shortening of these muscles (Tal'nov et al., 1997, 1999). The cited study demonstrated an exponential increase in elbow flexor EMG during these movements, which may correspond to the observed difference in derivatives in two length ranges, L2 and L1, covering the regions of their greater and lesser values, respectively (**Figure 4**). As with derivatives associated with a given relationship between force and length amplitudes, the distribution of their statistical parameters (both mean values ​​and variances) across the different test groups is clearly distinguished in these ranges. Provided that using symmetric set of the force changes including five patterns F(1.0), F(0.5), iso, F(-0.5), F(-1.0), one can speak about a general coincidence in change of the statistical characteristics $\frac{\partial E(t)}{\partial t}$ if to compare identical phases of movement (shortening or lengthening) in P1 and P2 tests, in which these phases have opposite order. This is most clearly shown in **Figure 5**, where their average values ​​are presented. In **Figure 6** the identical halves of the movement tests P1 and P2 are regrouped to compare action of the identical combination in the force and length changes. Following this procedure, similar dependencies of averaged derivatives $\bar{\frac{\partial E(t)}{\partial t}}$ in P1 and P2 movement tests can be compared with respect to different force amplitudes (**Figure 6AII**). Despite some natural differences in the dependencies obtained for the two test movements, this approach can be useful for identifying nonlinear effects in the system under study. At the very least, this method clearly reveals differences in the slopes of the corresponding dependencies across two muscle length ranges, L1 and L2.

**Figure 4:**
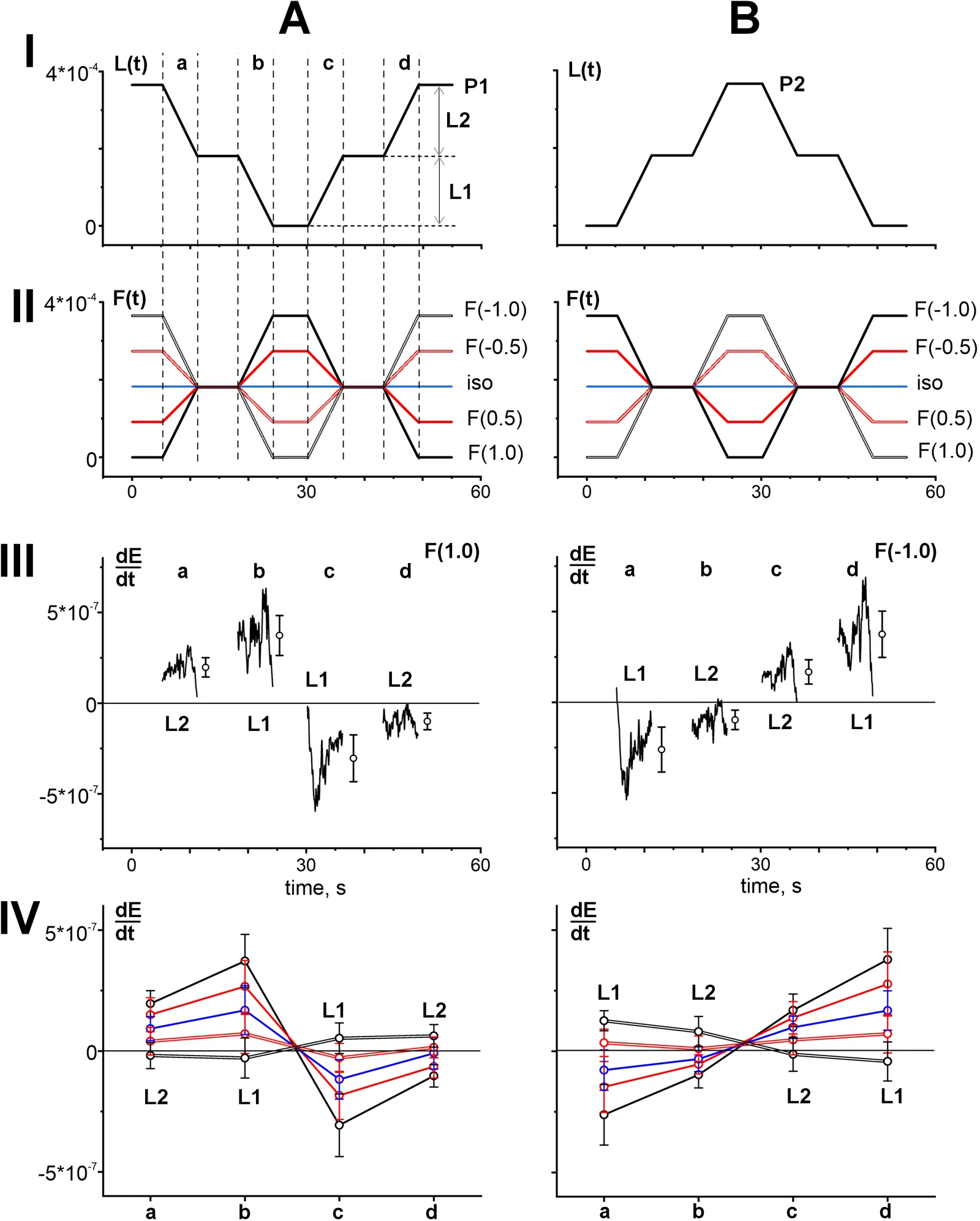
Determination of statistical characteristics (mean ± SD) of activation intensity derivatives. **(A, B)** Statistical characteristics are calculated for two test variants P1, P2 in time intervals a–d, during which muscle length and force change. Row III shows an example of responses and statistical characteristics recorded in tests with maximum changes in force directed oppositely to length changes; row IV presents statistical data for all tests; note the correspondence of color and line structure between rows II and IV.

**Figure 5:**
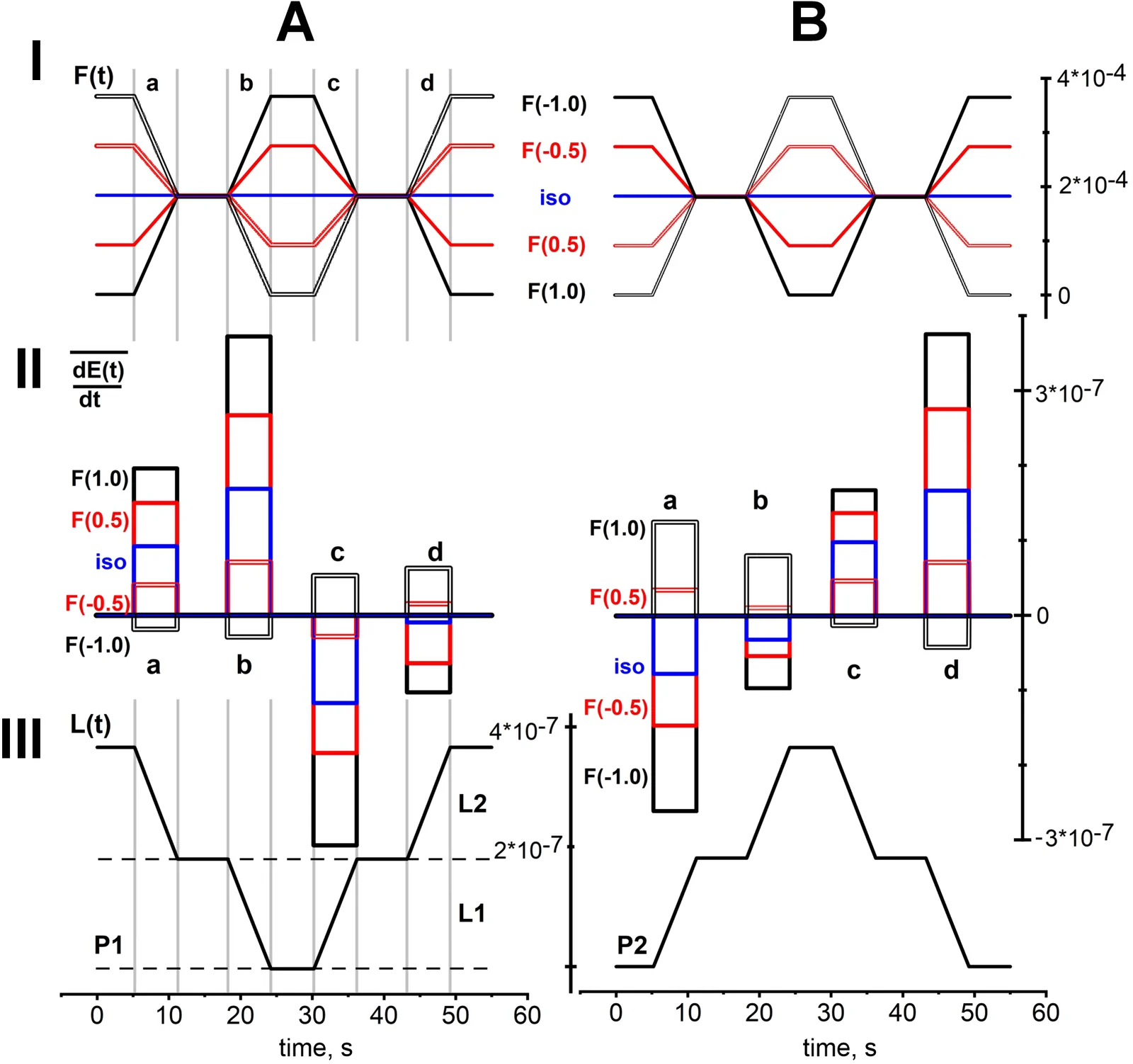
Visualization of the relationship between the averaged values ​​of the E(t) derivatives and the corresponding changes in force and length. **(A, B)** Comparison of the two movement tests P1 and P2; note the consistency of color and line structure between the graphs in rows I and II. In the second row, the blue bars represent reactions in the isotonic mode, whilst the red and black bars correspond to responses to intermediate and maximum amplitudes of force change.

**Figure 6:**
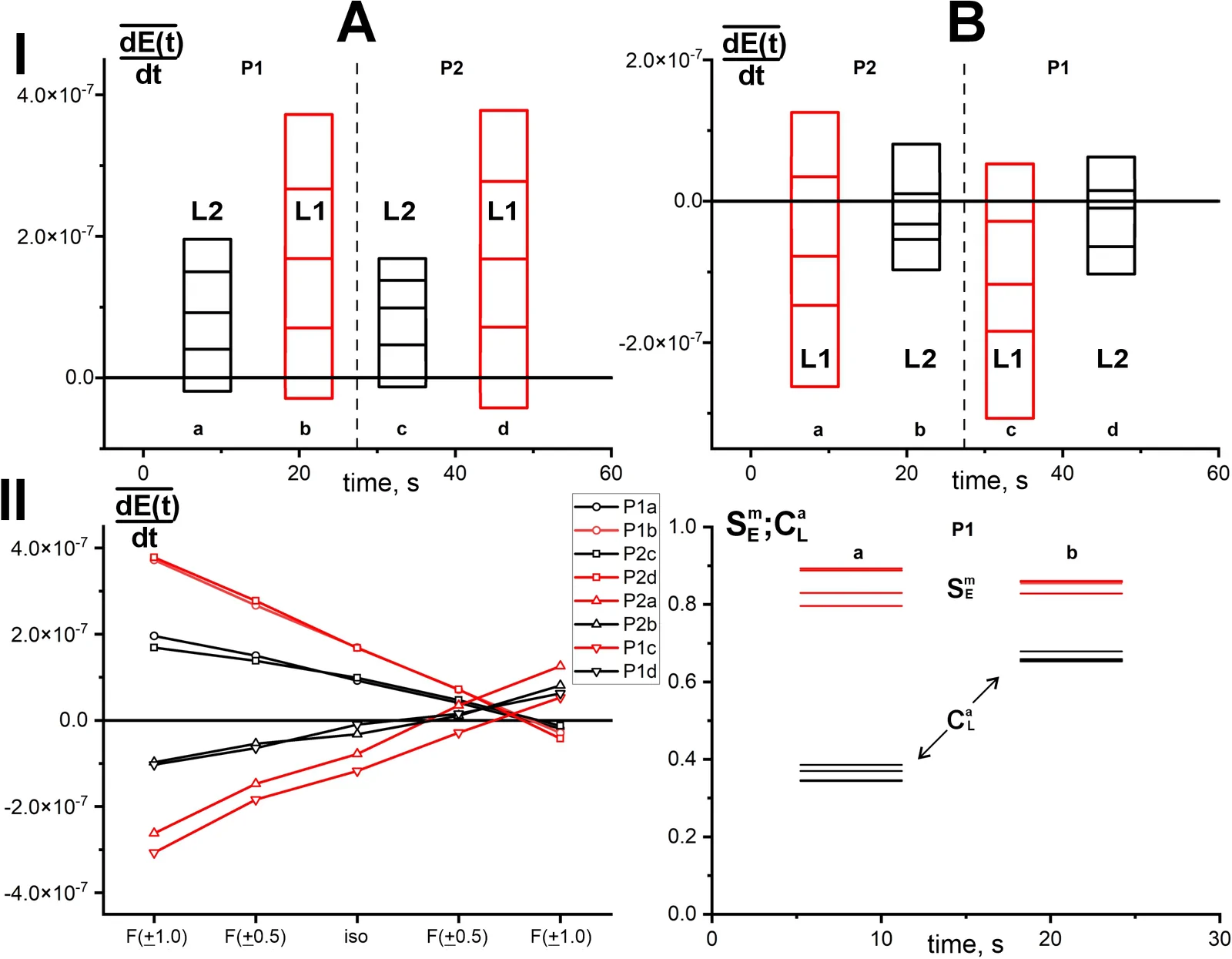
Joint influence of the mean derivatives of force and length on the mean derivative of activation intensity. **(A, B)** The mean activation intensity derivative plots in **Figure 5** are split in half in P1 and P2 movement tests and regrouped to compare them for the same movement directions (shortening in **AI**, lengthening in **BI**). The values ​​represented by the vertical bars in panels **AI**, **BI** are transferred to the graphs in panel **AII**, plotted as functions of the amplitude of force changes (note the color correspondence between these graphs and the bars). The red and black horizontal lines in panel **BII** represent the results of muscle stiffness and activation compliance calculations according to Equations 13, 14.

### 3.5 Determination of muscle stiffness and activation compliance with simultaneous changes in muscle force and length (inverse problem of T-modelling)

Muscle force and activation compliance, introduced by Equation 3 can be determined as follows. It can be firstly started with the isotonic regimen, simplifying Equation 8 for this case as follows:

(11) $${\left(\bar{\frac{\partial E}{\partial t}}\right)}_{iso}=-C_{L}^{a}S_{E}^{m}{\left(\bar{\frac{\partial L}{\partial t}}\right)}_{iso}$$

Then, based on Equation 11, it can be rewritten Equation 8 as follows:

(12) $${\left(\bar{\frac{\partial E}{\partial t}}\right)}_{F(\Delta )}=C_{L}^{a}{\left(\bar{\frac{\partial F}{\partial t}}\right)}_{F(\Delta )}+{\left(\bar{\frac{\partial E}{\partial t}}\right)}_{iso}$$

where: Δ=±1.0; ±0.5.

Equation 12 allows to directly define the activation compliance parameter:

(13) $$C_{L}^{a}=\frac{\left[{\left(\bar{\frac{\partial E}{\partial t}}\right)}_{F(\Delta )}-{\left(\bar{\frac{\partial E}{\partial t}}\right)}_{iso}\right]}{{\left(\bar{\frac{\partial F}{\partial t}}\right)}_{F(\Delta )}}$$

where: Δ=±1.0; ±0.5.

Finally, calculating the activation compliance using Equation 13 opens the way to calculating muscle stiffness using Equation 8:

(14) $$S_{E}^{m}=-\frac{{\left(\bar{\frac{\partial E}{\partial t}}\right)}_{iso}}{C_{L}^{a}{\left(\bar{\frac{\partial L}{\partial t}}\right)}_{iso}}$$

Attempts to determine muscle stiffness and activation compliance are presented in **Figure 6BII**; the consideration will be restricted by the more stable and predictable responses in the first half of the P1 movement trials. First, using Equation 13, two sets $C_{L}^{a}$ (black lines) are determined for time intervals a and b, each containing four values. These values ​​were then used to calculate two sets $S_{E}^{m}$ (red lines) using Equation 14.

The features of the $C_{L}^{a}$and $S_{E}^{m}$ positioning shown in **Figure 6BII** can likely be used to explain the observed differences in the slopes of the $\bar{\frac{\partial E(t)}{\partial t}}$ dependences plotted against force amplitudes (**Figure 6AII**). At the very least, it can be speculated that the apparent differences between the slopes of the corresponding pairs of black and red lines (**Figure 6AII**) relating to the data recorded in the L2 and L1 length ranges (**Figures 6AI, BI**) are most likely determined by the nearly twofold difference in activation compliance, whereas muscle stiffness remains virtually unchanged in these length ranges (**Figure 6BII**).

### 3.6 Slopes of the activation intensities during muscle force and length changes

The sequential process of piecewise linear approximation of activation intensity allows one to partition time intervals into more detailed regions where muscle force and length are changing, whereas for steady state intervals it is probably better to use their simplified form:

(15) $$\tilde{E(t)}=\int_{1}\bar{\frac{\partial E}{\partial t}}dt+\int_{a}C_{L}^{a}(\bar{\frac{\partial F}{\partial t}}-S_{E}^{m}\bar{\frac{\partial L}{\partial t}})dt+\int_{2}\bar{\frac{\partial E}{\partial t}}dt+\int_{b}C_{L}^{a}(\bar{\frac{\partial F}{\partial t}}-S_{E}^{m}\bar{\frac{\partial L}{\partial t}})dt+\ldots$$

The first type of integrals is determined in time intervals **1–5** where the force and length of muscles do not change; the second type, in time intervals a–d, is associated with the zones of their change (as shown in **Figure 1**). In this case, the endpoints of each previous integral create the initial conditions for the subsequent ones. Thus, the approximation curves in **Figure 7**, originally constructed using Equation 10, can be further analyzed in more detail using the second type integrals of Equation 15. For comparison of the real activation intensity curves with their linear approximations, they both are shown within the time intervals a – d in **Figure 7AI, BI**, whereas parts of linear approximations are plotted separately in row II of **Figure 7** in order to compare the coincident parts in P1 and P2 movement tests for different force amplitudes. When comparing the corresponding pairs of time ranges, a, c, related to P1 tests (**Figure 7AII**), and b, d, related to P2 tests (**Figure 7BII**), the fit lines show opposite directions of rotation during transitions from isotonic muscle contractions to changes in force directed opposite to (or coinciding with) changes in length. Starting from initial positions of the “isotonic” blue lines, in first case these lines are moving in counter-clockwise direction (single red and black lines), whereas the second movement type is a clockwise (double red and black lines). The slope of the fit lines increases as the muscle contraction force changes in the direction opposite to the length. Conversely, if the directions of force and length change coincide, the slope of the lines decreases. This behavior of the approximation lines is consistent with the difference relationship of the derivatives of force and length in the expressions of the integrands of the corresponding integrals in Equation 15. Moreover, it is precisely this difference that allows to pinpoint the equilibrium point in the opposing relationships between the two types of effects, force, and length, on the activation intensity, at which the influence of force begins to manifest itself predominantly as compared to the length changes. The equilibrium point can be determined to be somewhere between the maximum and half-maximum values ​​of the force changes in coinciding direction, where the sign of the slope of the corresponding fit lines changes to the opposite. It seems to be important that the sign inversion in the slopes of the fit lines is repeated in all time ranges for both test movements P1 and P2 (**Figures 7AII, BII**).

**Figure 7:**
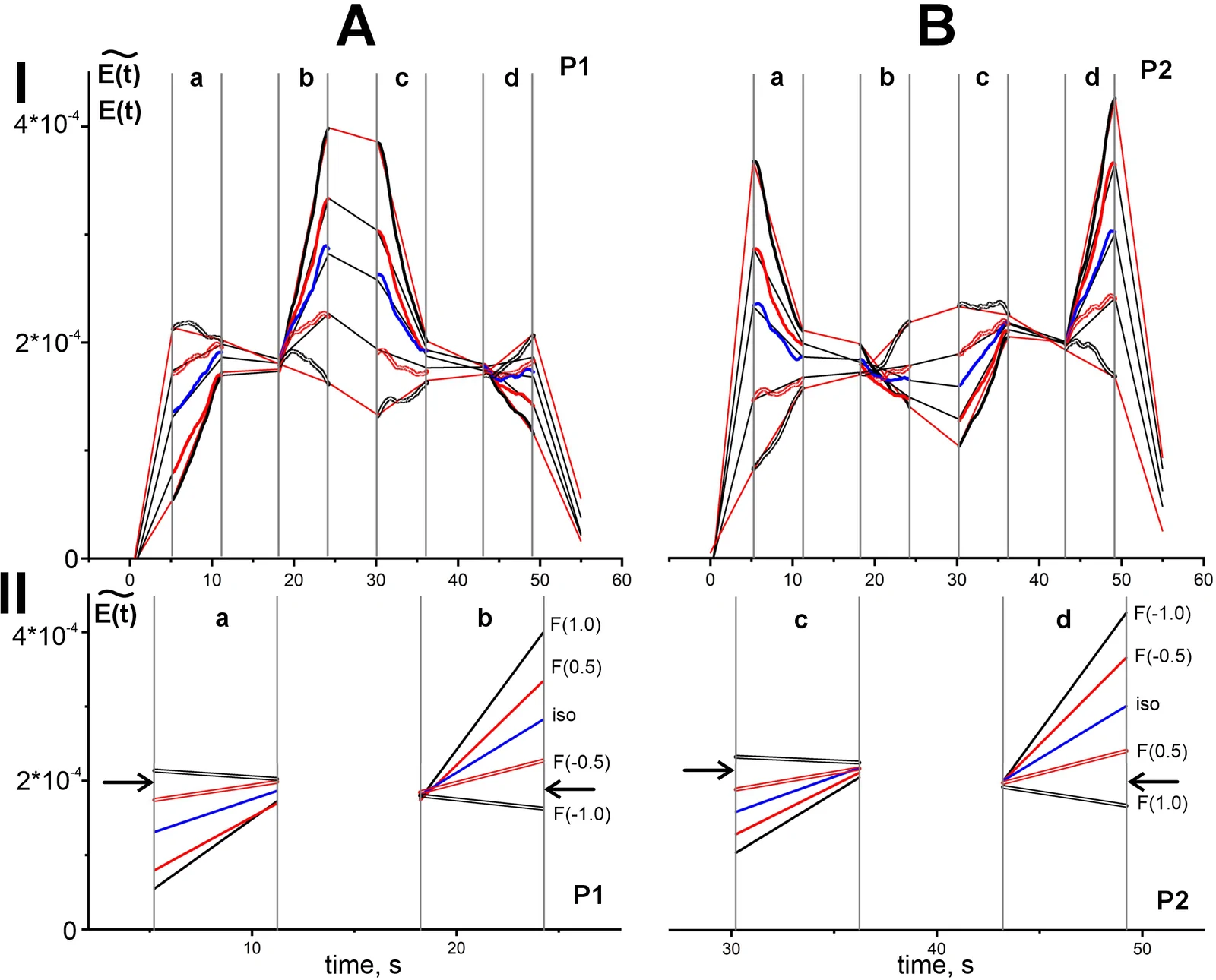
Differences in the slopes of the activation intensity approximations across various time ranges of simultaneous changes in muscle force and length. **(A, B)** Superposition of piecewise linear approximations obtained for five combinations of force changes and two movement test variants P1, P2. Respective sections of the averaged activation intensity curves are added in AI and BI panels over the approximation lines in the corresponding time ranges a-d. Note the correspondence between the color and line structure between E(t) curves **(AI, BI)** and their approximations $\tilde{E(t)}$ (**AII, BII**). The arrows in panels AII and BII show the expected amplitudes of force change, somewhere between F( ± 0.5) and F( ± 1.0), which equalize reactions to the length change.

The quality of the piecewise linear approximation using the T-model was statistically assessed using two parameters: *Reduced χ²* (Homer, 2018) and *COD R-square χ²* (Yin and Fan, 2001). The results are presented in **Table 1**. Five types of tests, differing in the direction and amplitude of force changes (rows in **Table 1**), were compared for two pairs of test sections that had identical programs of force and length changes within the P1 and P2 movement patterns. These sections are designated by the letters a, b (**Figure 7AI**) and c, d (**Figure 7BI**), respectively. The lowest value of the *COD* coefficient, equal to 0.9389, indicates that the T-model provides a reasonably good fit to the present experimental data. At the same time, it can be noted that the quality of the approximation seems to be better in the OP and isotony (ISO) tests, and somewhat worsens in both CP tests. It can be hypothesized that the above differences may be related to clearly visible differences in the amplitude of the corresponding EMG recordings. At least in part, the smaller amplitude of EMG signal oscillations may lead to an increased relative weight of nonlinear effects associated with the filtering procedures applied to row EMG recordings in this study. Conversely, the significant increase in the amplitude of EMG responses in both ISO and OP tests may reduce the aforementioned nonlinear distortions.

**Table 1:** Assessment of the quality of piecewise linear approximations of EMG intensity records (population averaging) by T-model.

Force – length direction relationship	Movement pattern			
	P1		P2	
	Reduced χ^2^	COD R-square	Reduced χ^2^	COD R-square
OP (max amplitude of force change)	1.4·10^-11^	0.9986	4.0·10^-11^	0.9956
OP (half-max amplitude of force change)	1.5·10^-11^	0.9973	2.2·10^-11^	0.9956
ISO (isotony force)	8.8·10^-12^	0.9957	3.5·10^-12^	0.9981
CP (half-max amplitude of force change)	7.8·10^-12^	0.9719	6.3·10^-12^	0.9718
CP (max amplitude of force change)	1.6·10^-11^	0.9389	1.3·10^-11^	0.9761

The quality of piecewise linear approximations is assessed for two pairs of similar motion sections, designated in **Figure 7** by the letters a, b (P1) and c, d (P2). Parameters of fitting goodness are defined by Reduced χ^2^ (reduced chi-squared, Homer, 2018), and COD R-square (R-square coefficient of determination, Yin, Fan, 2001). The number of approximated points consists 1202 in all cells.

The simultaneous presentation of the linear approximations of shortening and lengthening EMG reactions (**Figure 8**) can be used to consider possible differences in the hysteresis mechanisms for *opposing* (OP) and *coinciding* (CP) types of movements. Despite the natural differences between similar EMG responses in the P1 and P2 movement tests, a striking similarity can be observed when comparing their patterns in each of the length ranges, L1 and L2. It is important to note the difference between the pairs of shortening and lengthening lines in the OP and CP tests (the shortening lines are wider in all cases). In the CP pairs, the shortening lines predominantly extend upward, indicating their “normal” hysteresis in these pairs, that is, a higher activation intensity during shortening processes. Thus, mean amplitude of hysteresis difference between shortening and lengthening lines under conditions of maximal force equals (mean ± standard error mean) -6.47·10^-6^ ± 2.64·10^-6^ (P1, L1, OP), 2.24·10^-5^ ± 3.75·10^-7^ (P1, L1, CP), 2.37·10^-5^ ± 2.68·10^-6^ (P2, L1, OP), 6.09·10^-5^ ± 8.90·10^-7^ (P2, L1, CP). In contrast, such dependencies between these processes are often partially or completely disrupted in the OP pairs of lines. This is particularly noticeable in the L2 length range for maximum force amplitudes (black lines), where the “normal” hysteresis of the CP tests reverses direction in the OP tests (**Figures 8AI, BI**).

**Figure 8:**
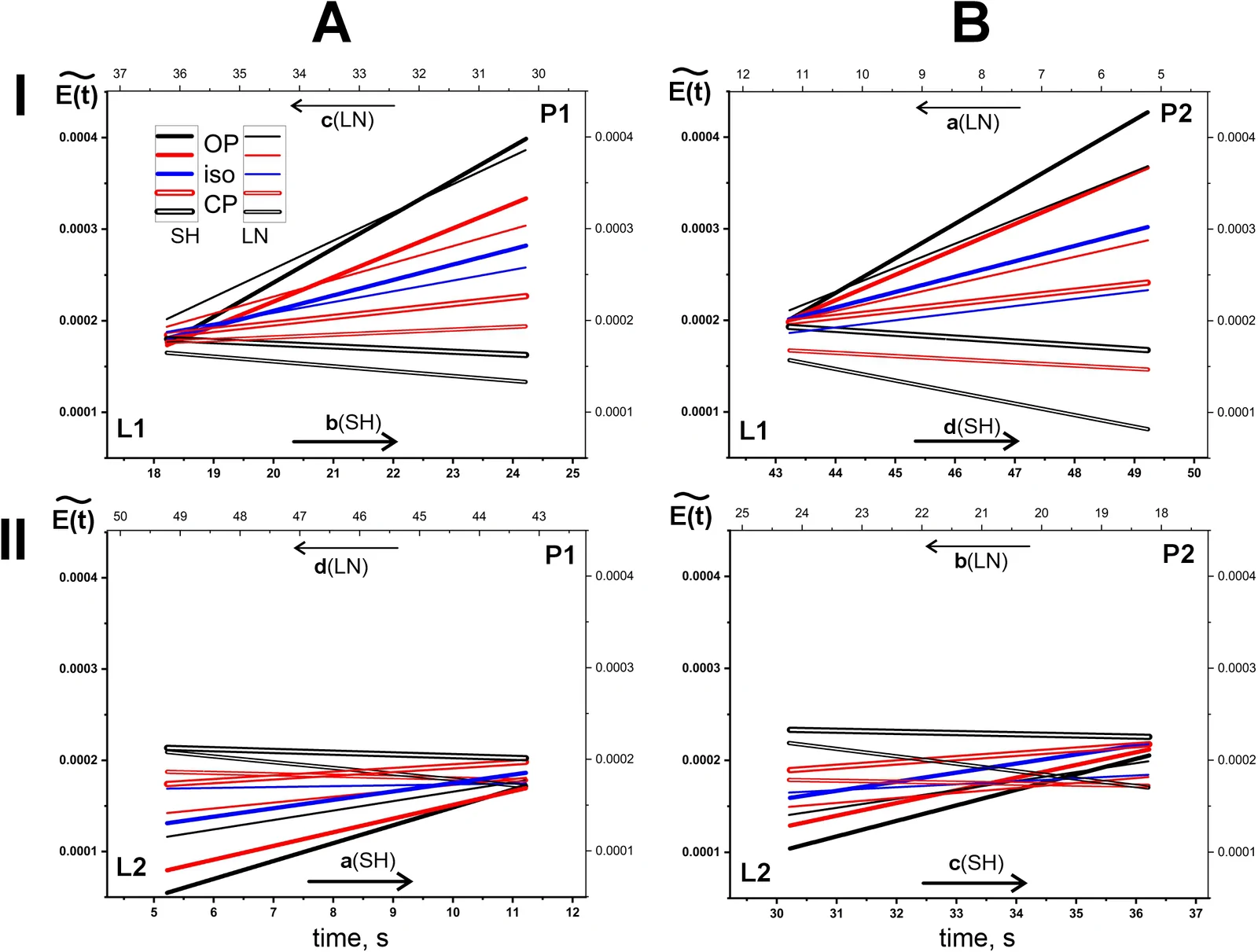
Hysteresis differences in the slopes of the activation intensity approximations across various time ranges of simultaneous changes in muscle force and length. **(A, B)** Comparison of piecewise linear fits between shortening (SH, thick lines) and lengthening (LN, thin lines) branches within the same length intervals (L1, L2) and during the same tests (P1, P2); the lengthening plots are presented in reverse time representation, shown in the scales at the top of the panels. Each panel shows five combinations of force changes, including one isotonic load (blue lines) and two pairs of force trajectories changing in either the opposing pattern (OP) or the coinciding pattern (CP) with respect to the length changes. Respective sections a-d signify corresponding time intervals within tests, where muscle lengths are changing (see **Figure 1**, line **I**). Note that for both pairs of CP traces (lines I and II) in both movement tests **(A, B)**, the lines corresponding to shortening movements (SH) predominantly pass above the lengthening ones (LN), whereas in other cases, including tests with isotonic loads, partial or even complete violations of the standard hysteresis relationship between shortening and lengthening activation intensities may be observed.

## 4 Discussion

The main objective of the study is to develop algorithms for predicting the intensity of activation in groups of closely synergistically interacting muscles when performing tasks that simultaneously change their force and length. For linear changes of these mechanical parameters of the muscle contraction, the nervous system in many cases should also generate linearly changing activation intensity. This linearity creates convenient conditions for EMG approximation, and, at least for the short intervals of parameter change used in the experimental study, about 4 s, it was found that using the averaged EMG derivatives over time in these intervals often gives an almost perfect approximation of their linearly increasing/decreasing portions (**Figure 2**). Equation 7, and Equation 8 including the basic elements of the present modeling describes important functional relationships between time derivatives of the functions under study: E, F, L. The difference between the force and length derivatives reflects the opposite nature of their effects on the control signal E(t). Over the range of force-length ratios and velocities used, almost regular stepwise changes in the derivatives of the average activation intensity can sometimes be observed; at least, this can be seen for time ranges a and b in **Figure 5AIII** and d in **Figure 5BIII**. This visual impression is confirmed by the linearity of the graphs of the average derivatives E(t), plotted relative to the amplitudes of the force changes (**Figure 6AII**).

In this and the previous study (Gorkovenko et al., 2026), the authors aimed to find approaches to understanding the mechanisms of hysteresis effects in real human hand movements. In order to analyze these mechanisms, it is necessary to consider different combinations of changes in muscle length, force, and activation intensity. The problem lies in the possible formal existence of different types of hysteresis effects, which could interact with each other in unpredictable ways. Traditionally, muscle hysteresis has typically been observed during sustained efferent activation as an increase in force in response to muscle lengthening and a decrease in force during muscle shortening. The most suitable experimental model for demonstrating muscle hysteresis is the steady-state activation of muscles by direct stimulation of the entire nerve or its separate filaments together with application of corresponding slow linear stretches and shortenings of the muscles. However, these methods can only detect length-dependent hysteresis; fewer common tests include the identification of activation-dependent hysteresis, when muscle length changes during isotonic exercise and movements are evoked by reciprocating frequency-modulated stimulation of the innervating efferent fibers (Kostyukov and Korchak, 1998; for a review, see Kostyukov, 2007). It turned out that the two types of hysteresis effects, depending on the input parameters, i.e. muscle length and activation intensity, can differ slightly from each other, and these differences mainly concern the initial sections of force change associated with the earliest phases of input signals. When length-dependent hysteresis exhibits virtually identical temporal characteristics of initial muscle stiffness (the so-called zones of the short-range stiffness) in both types of lengthening-shortening transitions, the activation-dependent hysteresis exhibits a dramatic difference in initial force changes during the increasing and decreasing phases of activation intensity. It was shown that the latency of the onset of muscle lengthening relative to the onset of a linear decrease in the rate of muscle nerve stimulation is significantly higher than the same parameter of muscle shortening caused by an increase in rate (Kostyukov and Korchak, 1998).

The pronounced asymmetry of the rate-dependent rise and drop in the muscle contraction forces may be considered as one of possible differences between two types of hysteresis. This study seems support an idea that these effects may interact. One can see in the present, much more complicated experimental paradigms of human experiments compared with traditional animal experiments, that the hysteresis effects can be evidently diminish in OP tests, remaining probably unchanged in CP tests (**Figure 8**). At least, it concerns of the linear approximation of the EMG reactions in the framework of the present model approach. However, one can assume a hypothetical mechanism for processes lying in the basis of the hysteresis effects. Earlier it had been proposed a mechanistic model for appearance of muscle hysteresis that might be associated with the dependence of the rate and/or intensity of mechanochemical processes on the direction of muscle length change (Kostyukov, 2007). It was put forward an assumption that muscle hysteresis can appear due to a difference in pure mechanical properties of two basic contractile myofilaments, providing the force generation, which predominantly include the actin and myosin contractile proteins. During muscle contractions the external change in muscle length above isometric value might cause a purely mechanical effect on the troponin-tropomyosin complex (TTC), which is known to play a crucial role in regulation of the contraction masking (Weber and Murray, 1973; Huxley, 1974). Assuming that the mechanical compliance of the actin helix is ​​higher than that of the TTC, an increase in tensile force should lead to displacement of the TTC into the actin double helix compared to isometric conditions, thereby increasing the efficiency of cross-link formation between the two types of myofilaments. During muscle unloading, the reverse process occurs, namely, the release of TTC from actin myofilaments, leading to additional steric blockage of the myosin binding sites. Such a model would apparently not contradict modern concepts of the molecular mechanisms of muscle contraction during forced stretching of active muscles. Titin, a giant multisegment protein that spans half the sarcomere and mechanically connects the Z-line and midregion of the myosin filament (Nishikawa, 2020), is currently being considered as a candidate structural element involved into the stretch-induced force enhancements (Herzog and Leonard, 2002; Leonard et al., 2010). Titin has been shown to be a complex muscle filament whose composition and geometry vary depending on muscle type (Tomalka, 2023), changing significantly across species (Lindstedt and Nishikawa, 2017). However, determining which interactions predominate between different titin segments and other filaments in the sarcomere has proven challenging.

In some of contemporary studies both two different branches of the hysteresis effects and their residual effects are considered independently of each other (Rassier and Herzog, 2002, 2004);. It is worth recalling that these studies have mostly focused on the molecular mechanisms underlying these phenomena. Notably, their findings were obtained from a series of studies conducted both on whole muscles (Raiteri et al, 2024) and on single myofibrils (Pun et al., 2010; Desai et al., 2026). It was shown in these experiments that hypotheses were proposed regarding the molecular mechanisms underlying the non-linear properties of muscle contraction: sarcomere length non-uniformity, a passive elastic element (i.e., titin), and an increase in the number of cross-bridges. None of the three hypotheses provide a full explanation, although none can be completely ruled out (Minozzo and Lira, 2013). Despite the undeniable importance of such studies, they generally do not consider muscle hysteresis as a general phenomenon associated with interconnected mechanisms of muscle contraction at the molecular level. On the other hand, attempts to find similar relationships can be found in the study of Walcott and Sun (Walcott and Sun, 2009).

Focusing solely on the molecular mechanisms underlying the nonlinear properties of muscle contraction may not lead to significant progress in understanding the mechanisms that control motor activity in humans and animals. Clearly, the central nervous system takes these nonlinear properties into account and utilizes them when organizing, planning, executing, and learning various movements. The present approach examines cyclic movements that closely resemble everyday activity. However, this method also has its natural limitations. Unfortunately, the present results do not allow to determine the specific molecular mechanisms underlying these nonlinear phenomena. The hysteresis effects in muscle contractions with relatively weak activation of a limited number of arm muscles may participate in generating motor commands to synergist muscles involved in more intense motor activity. Movement- and activation-dependent processes involved in the modification of muscle hysteresis appear to need to be considered, particularly in movement programs with a high degree of antagonist muscle involvement, such as walking and running, in which coactivation and reciprocal activation of antagonist muscles may play a crucial role (Minetti, 1994; Gorkovenko et al., 2012). In multi-joint, highly coordinated movements involving multiple limbs, such as walking, three partially independent types of muscle synergy are typically considered: kinematic synergy (Santello and Soechting, 2000; Thakur et al., 2008; Soechting and Flanders, 1997), force, or kinetic synergy (Grinyagin et al., 2005), and activation synergy (Weiss and Flanders, 2004; Castellini and van der Smagt, 2013; Bruton and O’Dwyer, 2018; Wang et al., 2013; Valero-Cuevas, 2000).

The present study suggests that interpreting muscle hysteresis solely in terms of residual force enhancement during trapezoidal lengthening tests may be insufficient without considering both the lengthening and shortening phases of movement. The results demonstrate that muscle hysteresis can vary substantially depending on the relative direction of changes in muscle length and activation intensity. In particular, the direction and magnitude of force changes relative to length changes can markedly modulate EMG hysteresis, revealing nonlinear effects that have received little attention in previous studies. These findings have important implications for the optimization of coordinated arm movement training and rehabilitation in individuals with musculoskeletal injuries or neurological disorders.

From a sports science perspective, the observed dependence of EMG hysteresis on the interaction between muscle force and length may contribute to a better understanding of neuromuscular adaptations during cyclic and repetitive movements. The proposed model may therefore assist in the development of training strategies aimed at improving movement efficiency, optimizing muscle loading, and reducing the risk of overuse injuries in athletes engaged in activities requiring repetitive upper-limb actions.

### 4.1 Limitations

A limitation of the present study is that dedicated experiments specifically designed to validate the proposed modeling approach could not be performed. The model was developed and evaluated using population-averaged EMG and muscle state parameter data obtained from the authors’ previously published experimental study (Gorkovenko et al., 2026). Although these data provided an appropriate basis for examining the proposed relationships, further experimental studies with protocols specifically designed to independently manipulate and quantify simultaneous coinciding and opposing changes in muscle force and length are required to validate the model at the individual-subject level and determine its generalizability.

### 4.2 Conclusions

A mathematical model is developed to characterize EMG responses of human elbow flexor muscles to *opposing* and *coinciding* changes in muscle length and force. The study provides algorithms for predicting activation intensity in synergistic muscle groups during motor tasks involving simultaneous changes in force and length. It is found that averaged EMG time derivatives over movement intervals provide a highly accurate approximation of the temporal dynamics of muscle activation. The model demonstrates that when force amplitude changes in the same direction as muscle length, EMG hysteresis amplitude increases, whereas changes in force in the opposite direction substantially reduce hysteresis effects, which can sometimes reach negative values.

## Data Availability

The raw data supporting the conclusions of this article will be made available by the authors, without undue reservation.
